# Kinematic, Dynamic, and Energy Characteristics of Diastolic Flow in the Left Ventricle

**DOI:** 10.1155/2015/701945

**Published:** 2015-08-31

**Authors:** Seyed Saeid Khalafvand, Tin-Kan Hung, Eddie Yin-Kwee Ng, Liang Zhong

**Affiliations:** ^1^School of Mechanical and Aerospace Engineering, Nanyang Technological University, Singapore 639798; ^2^Department of Bioengineering, University of Pittsburgh, Pittsburgh, PA 15261, USA; ^3^National Heart Research Institute of Singapore, National Heart Centre Singapore, 5 Hospital Drive, Singapore 169609; ^4^Duke-NUS Graduate Medical School Singapore, 8 College Road, Singapore 169857

## Abstract

Blood flow characteristics in the normal left ventricle are studied by using the magnetic resonance imaging, the Navier-Stokes equations, and the work-energy equation. Vortices produced during the mitral valve opening and closing are modeled in a two-dimensional analysis and correlated with temporal variations of the Reynolds number and pressure drop. Low shear stress and net pressures on the mitral valve are obtained for flow acceleration and deceleration. Bernoulli energy flux delivered to blood from ventricular dilation is practically balanced by the energy influx and the rate change of kinetic energy in the ventricle. The rates of work done by shear and energy dissipation are small. The dynamic and energy characteristics of the 2D results are comparable to those of a 3D model.

## 1. Introduction

Analysis of blood flow in the left ventricle is of fundamental interest in studying cardiac function and dysfunction. Using magnetic resonance velocity mapping, Kim et al. [1] reported a large counterclockwise vortex in the ventricle during diastole. Kilner et al. [2] indicated flow patterns in the normal LV avoiding excessive dissipation of energy to facilitate an efficient ejection of blood. Asymmetric vortices appearing with anterior and posterior mitral leaflets were discussed by Ebbers et al. [3]. Other significant two-dimensional (2D) and three-dimensional (3D) mathematical models for inflow to the ventricle were reported by several groups [4–9]. Using a prolate spheroid model, Domenichini et al. [10] stated that flow patterns in normal heart were optimal in terms of minimal energy dissipation.

Numerical modeling of flow in LV was classified into three types [11]: geometry-prescribed CFD methods [12–16], immerse boundary (IB) methods [17–19], and fluid structure interaction (FSI) methods [11, 20, 21]. A geometry-prescribed method employs the moving wall as a boundary condition, while the FSI methods couple the equations of motion for fluid and myocardium. The latter is a very challenging task because of the geometry and material properties of valve leaflets. Saber et al. [12, 13] used combination of MRI and CFD for flow simulation and obtained a counter clockwise vortex for diastolic flow in the ventricle. Long et al. [14, 15] investigated the influence of boundary motion to flow patterns and reported a main counter clockwise vortex in normal hearts. Schenkel et al. [16] modeled time-dependent mitral and aortic orifices without including valve leaflet movements and reported asymmetry vortices, isosurfaces, and schematic flow structure for mitral flow. All these studies were made for different ventricles and could not be considered as an alternative to one another [16, 22].

Using 2D velocities obtained from phase-contrast MR imaging, Thompson and McVeigh [23] calculated pressure drops in LV. The results were validated in a dog model and in a pulsatile flow phantom with high-fidelity pressure transducers. Yotti et al. [24] obtained pressure difference between the ventricle apex and the outflow tract from postprocessing color Doppler M-mode images. Garcia et al. [25] showed 2D velocity measurement from Doppler-echocardiography with assumption that the large scales of the flow are approximately 2D in an LV plane of interest. The velocity component normal to ultrasound beams was estimated from the continuity equation. Uejima et al. [26] reported an echocardiographic method to show vortex flow pattern in the ventricle. Faludi et al. [27] discussed vortex formations in healthy ventricles, the effect of prosthetic valves on flow patterns, and low resolutions of echocardiographic 3D imaging technology. The onset mitral flow patterns were obtained by Eriksson et al. [28] using path lines traced for 25 msec. Charonko et al. [29] employed 2D phase-contrast MRI to calculate the temporal variation of pressure drop with the mitral flow velocity and discuss normal LV filling with vortices. The kinetic energy of inflow was calculated for 12 subjects. Using Doppler-echocardiography data to obtain 2D velocity field, Hendabadi et al. [30] reported a trajectory-based computation of blood transport patterns for assessments of stasis in the ventricle. Quantification of ventricular contraction was studied by Hung et al. [31] using kinetic energy flux calculated from velocity vectors of normal and dyssynchrony echocardiograms.

The present study is to relate kinematic, dynamic, and energy characteristics of inflow to a normal left ventricle. Because of rapid flow acceleration and deceleration with mitral valve motion, the 3D effect is relatively small in comparison with the longitudinal flow. The main feature of inflow can be captured and learned by using 2D finite volumes and MRI data of cardiac contraction and dilation. The effect of mitral valve motion on pressure drop is studied by comparing cases with and without modeling mitral leaflet motions. The detailed flow patterns indicate momentum transfer in the rapid curvilinear flow produced by ventricular dilation and reveal alteration in boundary layer, vortices, shear stress, pressure variations, and net pressure on valve leaflets. The flow process is continued by the ventricular contraction and the results are reported by Hung et al. [32]. The work-energy equation is employed to study energy transfer from wall motion to blood flow during diastole. For the normal case, the rate of work done by shear and the energy dissipation are small. The results are in agreement with the optimal flow in the ventricle reported by Kilner et al. [2] and Domenichini et al. [10].

## 2. Computational and MRI Approach

**Figure 1:**
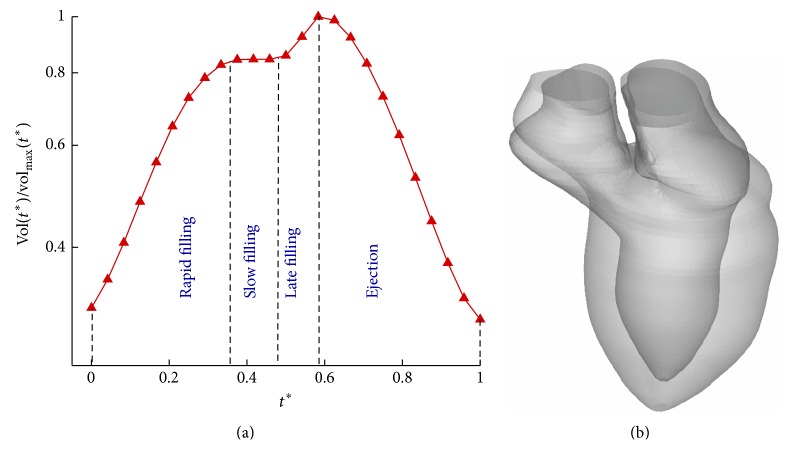
(a) Temporal variation of the left ventricle volume, vol(*t* ^*∗*^); *t* ^*∗*^ = 0 for the onset diastole and *t* ^*∗*^ = 1 for the end systole. (b) The change of geometry from end systole to end diastole.

MRI scanning was performed for a healthy adult on a 1.5T Siemens scanner (Avanto, Siemens Medical Solutions, Erlangen) using the steady-state-free precession cine gradient echo sequences. The data were acquired from 2-chamber, 4-chamber, and short-axis planes of the left ventricle using 12–14 equidistant slices. They were utilized for 3D reconstruction of movements of the left ventricle and atrium. The end-systolic and end-diastolic volumes are, respectively, 48.8 and 162.5 mL, producing a stroke volume of 113.7 mL and an ejection fraction of 70%. To simulate blood flow, 25 frames of LV endocardial walls were obtained from the MRI during one cardiac cycle. The temporal variation of ventricle volume, vol(*t* ^*∗*^), is shown in Figure 1(a) in which the cardiac period, *T* = 0.88 seconds, is used to define the dimensionless time, *t* ^*∗*^ = *t*/*T*, and vol_max_ is equal to the end-diastolic volume. The change of geometry from end systole to end diastole is shown in Figure 1(b).

Blood flow in large arteries can be treated as homogeneous Newtonian fluid of density 1050 kg/m^3^ and dynamic viscosity of 0.00316 Pa·sec. An arbitrary Lagrangian-Eulerian (ALE) formulation of the Navier-Stokes equations can be expressed as [33]

(1) ∂∂t∫Aρv→dA+∫sρv→v→−v→b·n→ds=−∫spI·n→ds+∫sτ·n→ds,

where $\vec{v}$ is the velocity vector, ${\vec{v}}_{b}$ is the local velocity along the boundary *s*, *p* is the pressure, **I** is the unit tensor, $\vec{n}$ is the unit normal vector, and **τ** is the viscous stress tensor. The integral form of two-dimensional continuity equation is

(2) $$\begin{array}{c} \frac{\partial }{\partial t}\int_{A}\rho dx\;dy+\int_{s}\rho \left(\;\vec{v}-{\vec{v}}_{b}\;\right)\cdot \vec{n}ds=0. \end{array}$$

The moving boundaries are determined from MRI data, providing velocities on the wall and mitral leaflets for computation. The Navier-Stokes equations for flow with moving meshes were solved using a finite volume CFD solver: ANSYS Fluent 14 (ANSYS, Inc.). A time-dependent uniform velocity profile is prescribed at the atrium inlet for modeling inflow to the mitral valve with the aortic valve closed. At each time step, the ventricle motion and mitral valve movements were implemented with the user defined functions (UDFs). For the 2D case, long axis images were used for the analysis and the mitral valve movements are derived from MRI data. Before the periodic flow was obtained, a grid dependency study was conducted for five cases with number of triangular cells increasing from 6000 to 9000, 13500, 20250, and 30375. The test results showed that the flow patterns became the same when the number reached 20250. This number was used and the grid was monitored, and remeshing method in ANSYS Fluent was applied when the grid quality was poor. The criterion for grid quality required the maximum value of face skew angle below 40 degrees. The grid convergence index (GCI) was used for assessing grid invariant solution [34]. The pressure implicit method with splitting of operators (PISO) algorithm [35] and a second-order upwind scheme were employed. Also, the Courant number criterion was satisfied; it resulted in 1800 time steps for one cardiac period (0.88 second) flow simulation. The results of diastolic flow in the left ventricle are presented in this paper.

## 3. Results and Discussions

**Figure 2:**
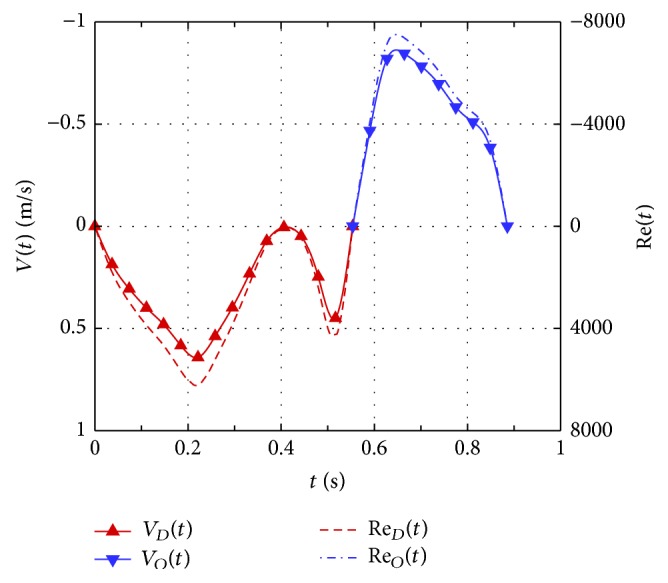
Time variations of inlet velocity *V*_*D*_(*t*) and the associated Reynolds numbers Re_*D*_(*t*) for diastolic flow. *V*_*O*_(*t*) and Re_*O*_(*t*) for systolic flow.

**Figure 3:**
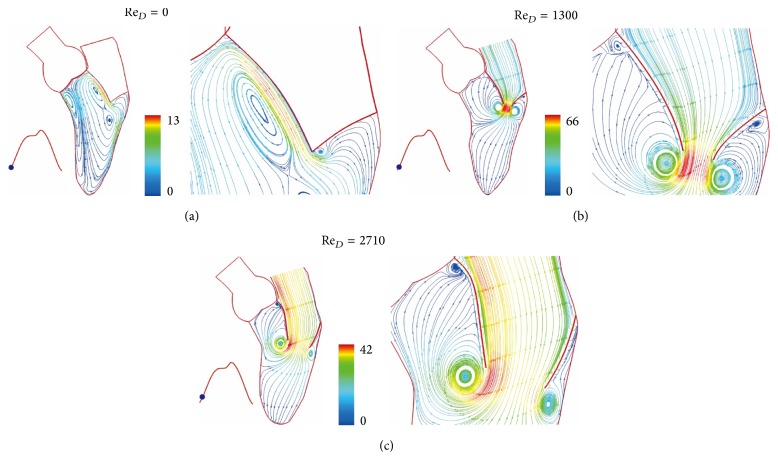
Streamlines at (a) *t* = 0 sec, (b) *t* = 0.0185 sec, and (c) *t* = 0.0738 sec. Streamlines colored by velocity (cm/sec).

The computation was initiated at the onset diastole and periodic solutions were obtained after 4 cycles of diastolic and systolic flow simulation.  Figure 2 shows the temporal variations of velocity *V*_*D*_(*t*) at the atrium inlet for diastolic flow and *V*_*O*_(*t*) at the outlet of the sinus of Valsalva for systole; they are plotted as negative for inflow and positive for outflow, respectively. The nonlinear pulsating flow processes cannot be effectively characterized by the mean Reynolds number with several frequency parameters but by the time-dependent Reynolds numbers (see Figure 2) for the diastole and systole [36]:

(3a) ReDt=ρVDtD1tμ,

(3b) ReOt=ρVOtD2tμ

in which *D*_1_(*t*) and *D*_2_(*t*) are the inlet and outlet diameters. Notice that using the time-dependent diameters leads to expressing the instantaneous flow rate by Re^2^ *μ* ^2^ *π*/4*ρ* ^2^ *V*. Correlation of Re_*D*_(*t*) with pressure drop is an effective way to present nonlinear pulsating flow processes.  Figure 3(a) shows ventricular vortices at the end systole with the aortic and mitral valves closed and the ventricle momentarily stationary. An early phase of flow acceleration is shown in Figure 3(b) for the Reynolds number (Re_*D*_) rising from zero to 1300 in 0.0185 seconds. The suction and momentum produced by wall dilation disperse immediately end-systolic vortices in Figure 3(a) and produce a pair of asymmetric vortices with mitral leaflets being opened by the inflow. Due to curvature effect, high momentum appears near the anterior leaflet. The streamlines near the leaflet tip contribute momentum to its vortex, while the other streamlines from the leaflet are pushed backward by momentum from outer region of the vortex, producing a small counter rotating vortex at the leaflet root. On the atrial side, the leaflet momentum interrupts the transient boundary layer, resulting in a sharp turn of streamlines towards the moving boundary. Due to mesh size effect on contour plots, streamlines near the leaflet appear parallel instead of slightly tapered.

**Figure 4:**
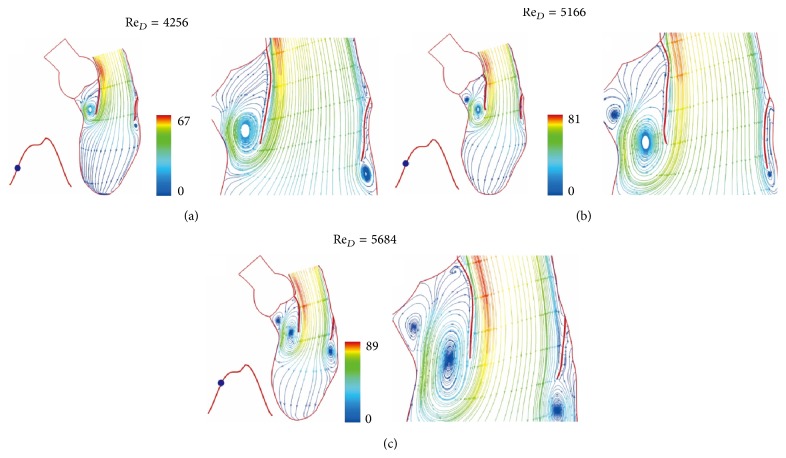
Streamlines at (a) *t* = 0.1476 sec, (b) *t* = 0.1845 sec, and (c) *t* = 0.2214 sec. Streamlines colored by velocity (cm/sec).

**Figure 5:**
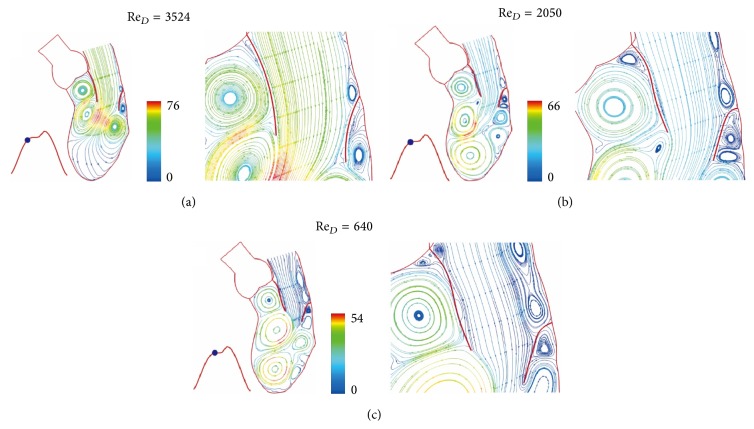
Streamlines at (a) *t* = 0.2952 sec, (b) *t* = 0.3321 sec, and (c) *t* = 0.369 sec. Streamlines colored by velocity (cm/sec).

**Figure 6:**
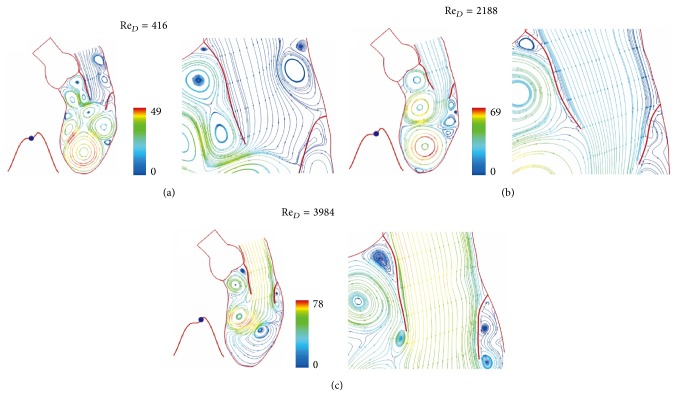
Streamlines at (a) *t* = 0.4428 sec, (b) *t* = 0.4797 sec, and (c) *t* = 0.5166 sec. Streamlines colored by velocity (cm/sec).

**Figure 7:**
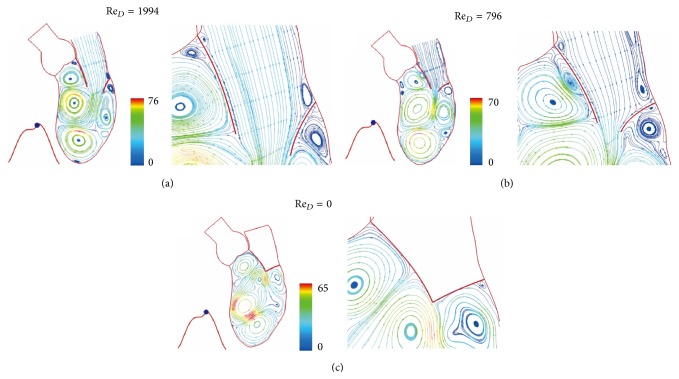
Streamlines at (a) *t* = 0.5289 sec, (b) *t* = 0.5412 sec, and (c) *t* = 0.5535 sec. Streamlines colored by velocity (cm/sec).

The color scale 66 in Figure 3(b) is for velocity scale of 66 cm/sec. Figures 3(c) and 4 show the continued flow acceleration when Re_*D*_ reaches 2710, 4256, 5166, and 5684. Because the ventricle dilation includes an upward movement of the closed aortic valve, the streamlines in this region move with the valve. Similar to laminar flow in a conduit expansion reported by Macagno and Hung [37], the zone of vortices provides a smooth pathway for blood flowing to the ventricle. As the rate of ventricular dilation decreases with elastic recoil of cardiac contraction, the flow begins to decelerate. A rapid growth of vortices appears in Figure 5(a) as Re_*D*_  reduces from 5684 to 3524 in 0.0738 seconds; it indicates momentum transfer from the main flow to vortices during deceleration [36]. More vortices are generated for momentum balance when Re_*D*_ drops to 2050, 640, and 416 (Figures 5 and 6(a)). Although the Reynolds number decreases drastically, velocities in the ventricle do not, reflecting small viscous effect in this rapid flow deceleration. This phenomenon correlates well with small energy loss shown in Figure 11. The momentum transfer produces a strong vortex near the apex when Re_*D*_ = 416. Before the complete closure of mitral leaflets, the ventricle dilation and inflow are assisted by atrial contraction. Increase in vortex momentum and reopening of the mitral valve are demonstrated for Re_*D*_ increasing to 2188 and 3984 in Figures 6(b) and 6(c).  Figure 7 portrays the final flow deceleration for the mitral valve closure. On the ventricular side the vortex momentum moves with valve closure which also pushes inflow to the ventricle. They are indicated by the streamlines moving with the leaflets for Re_*D*_ = 1994 and 796. These detailed flow characteristics reflect the capability of combining CFD with MRI data for blood flow with moving boundary. When the inflow vanishes at the end diastole (Re_*D*_ = 0), the ventricle is occupied by vortices. The formation of these vortices is simply for momentum balance at the end diastole. In the absence of end-diastolic atrial contraction, the stroke volume is likely to reduce unless the ventricular dilation is stronger.

**Figure 8:**
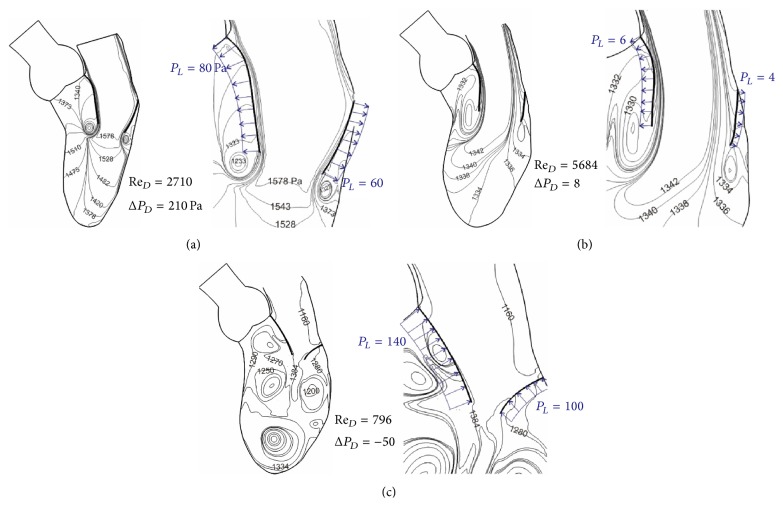
Pressure contours and net pressure on the mitral valve leaflets for (a) rapid filling, (b) for peak of inlet velocity, and (c) during closing of mitral valve.

**Figure 9:**
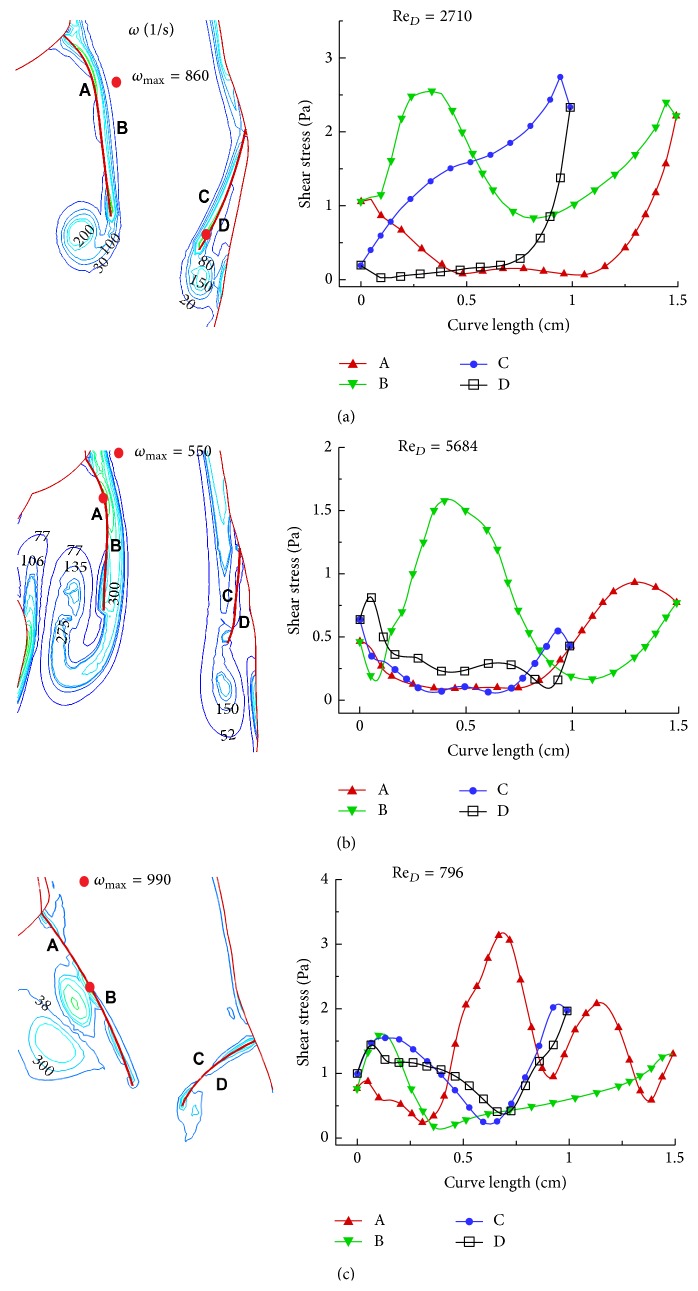
Vorticity and shear stress distribution of the flow past the mitral valve for (a) rapid filling, (b) for peak of inlet velocity, and (c) during closing of mitral valve.

Dynamic characteristics are demonstrated by pressure contours in Figure 8(a) for Re_*D*_ = 2710 during valve opening. The pressure drop between the center of the mitral annulus and the apex is indicated in the figure by Δ*P*_*D*_ = 210 Pa (1.6 mm Hg). The tip of tongue-shaped contours (e.g., marked by 1420 and 1378 Pa) correlates with streamlines (for similar velocities) in the region. The contour plot is based on a reference pressure of 1333 Pa (10 mm Hg) at the apex. The centrifugal force effect is reflected by low pressure at the center of strong vortex. Also shown in the figure are net pressures, *P*_*L*_ = 60 and 80 Pa, on mitral leaflets; they are related to the valve motion. The tongue-shaped pressure contours for Re_*D*_ = 5684 shown in Figure 8(b) are due to high momentum near the anterior leaflet. The small pressure drop (Δ*P*_*D*_ = 8 Pa) at this instant is related to diminishing flow acceleration. The net pressures (*P*_*L*_ = 4 and 6 Pa) on mitral leaflets correspond to valve in a fully open position; it is about 7% of that during the rapid opening phase (see Figure 8(a)). Adverse net pressures on leaflets appear in Figure 8(c) for Re_*D*_ = 796 with Δ*P*_*D*_ = −50 Pa. They are associated with the rapid flow deceleration and valve closure. Further dynamic characteristics can be seen from shear distributions on both sides of mitral leaflets (Figure 9). During the mitral valve opening phase shear stresses on the atrial side of leaflets are higher than those on the ventricular side. Because of leaflet motion, shear stresses are rather small and relate with vorticity (*ω*). The maximum vorticity (*ω*_max_) listed in the figure can be compared with wall vorticity (=620 sec^−1^) of the Poiseuille flow for the median Reynolds number of 2842.

**Figure 10:**
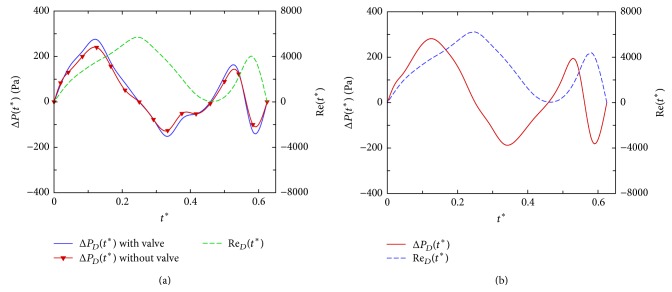
Time variation of inflow Reynolds number Re_*D*_(*t* ^*∗*^) and the pressure drop Δ*P*_*D*_(*t* ^*∗*^) for (a) 2D modelling of LV with and without mitral valve leaflets. (b) 3D modelling of LV.

Figure 10(a) shows the time variation of the Reynolds number (Re_*D*_) with pressure difference Δ*P*_*D*_ between the mitral annulus and the apex. They are due to ventricular dilatation produced by elastic energy stored in myocardium during contraction. Because of inertial effect, the peak flow is lag behind the maximum pressure drop which is in phase with the flow acceleration. Decreasing Re_*D*_ correlates well with adverse pressure drop. Also shown in this figure is the pressure drop for a case without modeling mitral leaflets. Small differences in pressure drop between cases with and without mitral leaflets indicate that a normal valve motion would not induce much of flow resistance. Justification of using 2D models can be made by similar pressure-flow curves of a 3D model shown in Figure 10(b) with the same inflow velocity *V*_*D*_(*t* ^*∗*^) of the 2D model. The pressure drops for the 2D and 3D models are comparable though the latter does not include the mitral valve motion.

**Figure 11:**
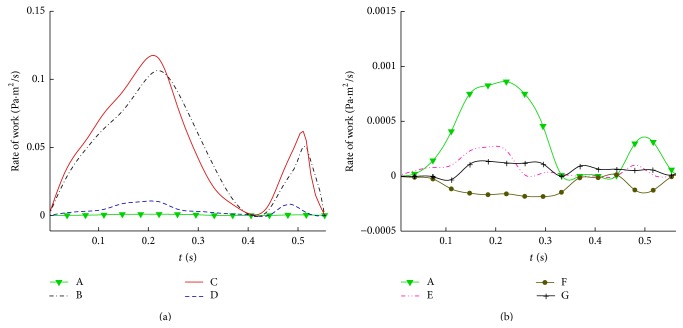
Rate of energy transfer of 2D model of diastolic flow in the left ventricle: curve A: kinetic energy flux on the wall, B − A: rate of work done by pressure on the wall, C − B: rate change of kinetic energy, C − D: rate of work by pressure at the outlet, D − E: kinetic energy influx, E − F: rate of work done by shear on the wall, and G − F: rate of energy dissipation.

The fluid dynamics of cardiac pumping can be further studied by using an integral form of the work-energy equation [37, 38]:

(4) ∫WρV22VNdl+∫WpVNdl+∬ρ2∂V2∂tdx dy−∫mpmVmdl−∫mρVm22Vmdl−∫W+mV→·τ→dl+μ∬2∂u∂x2+2∂v∂y2+∂u∂y+∂v∂x2dx dy=0

in which (*u*, *v*, *w*) are the velocity components in the Cartesian coordinates, *V*_*m*_ and *p*_*m*_ are the velocity and pressure across the mitral annulus, *V*_*N*_ is the normal velocity on the ventricle, and *dℓ* is the incremental line integral. The first five integrals are, respectively, the kinetic energy flux delivered to blood from the ventricle, the rate of work done by pressure on the wall, the rate of change of kinetic energy in the ventricle, the rate of work done by pressure across the mitral annulus, and the associated kinetic energy flux. The last two integrals are the rate of work done by shear and the rate of dissipation of energy, respectively. Notice that all the energy terms are positive; the sign for each term of (4) is related to the out flux and influx of energy. Curve A in Figure 11(a) is the kinetic energy flux delivered to blood from the ventricle during dilatation. It is amplified by about 100 times in Figure 11(b) for comparison with viscous terms in (4). The distance between curves B and A is the rate of work done by pressure on the wall. The gap between curves C and B represents the rate change of kinetic energy in the ventricle. It is positive when curve C is higher than curve B, otherwise, negative. The distance between curves C and D is the rate of work done by pressure across the mitral annulus. Since time variations of pressure are not known, calculation of the work done by pressure is based on an estimated reference pressure (1333 Pa or 10 mmHg) at the apex. The work done by pressure can be corrected by adding Δ*p*_1_(*t*)*V*_*D*_(*t*)*A*_*D*_(*t*) if Δ*p*_1_(*t*) = *p*_*A*_(*t*) − 1333 Pa, *p*_*A*_(*t*) is the actual pressure at the apex, and *A*_*D*_(*t*) is the cross-sectional area of the mitral section.

**Figure 12:**
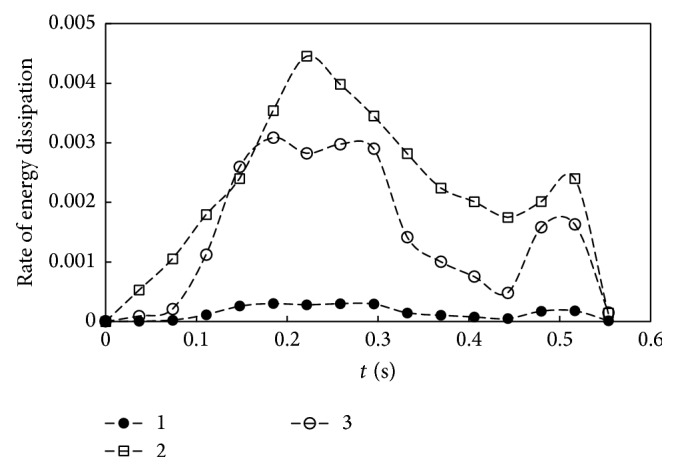
Comparison of the rate of energy dissipation between the 2D model (curve 1) and the 3D model (cure 2); curve 3 is the 2D results multiplied by the ratio between 3D and 2D flow rates.

The kinetic energy influx at the mitral section is indicated by the difference between curves D and E. It is about 13 times higher than that of kinetic energy flux from the ventricular wall (see curve A in Figure 11(b)). Notice that curve E is the sum of Bernoulli energy flux on the ventricle wall, the influx across the mitral annulus, and the rate change of kinetic energy in the ventricle. The value of E is very small, reflecting that the Bernoulli energy produced by ventricular dilation is practically conservative during the normal filling phase of blood to the ventricle. The gap between curves E and F is the rate of work done by shear stress on the wall. This small amount of energy is approximately equal to the rate of energy dissipation indicated by the spacing between curves G and F. All the aforementioned terms and curves are arranged so that curve G represents the sum of the left hand side of (4); it should be zero. The small values shown by curve G indicate the insignificant numerical residues of energy balance. The balance also reflects that the velocity and pressure fields obtained from CFD are quite accurate. In other words, the momentum balance achieved by the Navier-Stokes equations is well checked by energy balance of (4). Similar results are obtained for the 3D model; they will be reported separately.  Figure 12 compares the dissipation of energy between the 2D (curve 1) and 3D (curve 2) models. Curve 3 represents the results of the 2D case multiplied by the ratio of flow rates between 3D and 2D cases. It is lower than curve 2, indicating the effect of spiral flow on energy loss for the 3D case. Notice that the rate of kinetic energy flux on the wall can be directly calculated from the velocity of wall motion. This quantity was demonstrated as a useful index for quantification of echocardiograms with or without dyssynchrony [31]. The velocity vectors of echocardiogram are much easier to obtain then to reconstruct the LV motion from MRI data and conduct a CFD analysis. However the goal of this study is towards a comprehensive analysis of the dynamic and energy characteristics of blood flow produced by the left ventricle. Further quantification of energy parameters can be indicated by time integral of each energy term in (4). For example, the work done by pressure on the ventricle during dilation is the time integration of the area between curves B and A in Figure 11. Clearly, a physiological quantification of energy flux delivered from the ventricular chamber dilation requires 3D computational results.

## 4. Conclusions

Fluid dynamic characteristics of blood flow in the left ventricle are obtained by using 2D CFD with MRI data of a normal adult. The flow patterns are dominated by ventricular dilation and flow induced mitral valve movements. Generation and growth of vortices correlate well with flow acceleration and deceleration and mitral valve motion. They are solely for momentum balance and energy transfer from ventricle dilation to the curvilinear inflow of blood. Boundary layer and high shear stress do not develop on moving leaflets and ventricle. The work done by viscous stresses and dissipation of energy are small for normal diastolic flow. The energy loss is about 2% of the kinetic energy influx to the ventricle and is almost balanced with work done by viscous stresses. The Bernoulli energy flux from the ventricle dilatation to blood flow is practically balanced by the energy flux across the mitral annulus and the rate change of kinetic energy in the ventricle. In other words, the Bernoulli energy is conservative, indicating an optimal transport of blood from the left atrium to the ventricle. The dynamic and energy transfer characteristics obtained in the 2D model are in agreement with those of the 3D model. Similar dynamic and energy transfer characteristics were identified for the ejection phase of cardiac pumping of the left ventricle [32].
